# New Medical Journals

**Published:** 1845-09

**Authors:** 


### NEW MEDICAL JOURNALS.

We have received several numbers of the *Buffalo Medical Journal*, a young contemporary, which we should have noticed in our last number if it had not escaped our memory at the time. The editor is Austin B. Flint, M.D., lately a professor in the Rush Medical College, and a writer whose name is not unknown to our readers. It gives evidence of judgment, vigor, and taste in its editorial conduct, and will prove, we doubt not, a valuable accession to our periodical literature. The physicians of the region of country to whom it is especially offered should see to it that it is properly encouraged. The dissemination of journals of medicine widely through the country is one of the means by which we may hope to see the profession raised to higher dignity and augmented usefulness. The man of talents and learning, such as the editor of the Buffalo Journal possesses, who devotes his time and energies to the diffusion of sound science through the pages of such a periodical, ought to be considered a professional benefactor.

Professors Harrison and Carpenter, of New Orleans, propose adding to the number of medical journals in the South by the publication of a second one in that city. It is to be called the *Louisiana Medical and Surgical Journal,* and in the hands of editors of so much ability will take a high rank among its contemporaries. It has our most cordial wishes, and on the strength of what we know of its projectors we commend it heartily to the favor of our readers.

